# Identification of Ferroptosis-Related Genes as Biomarkers for Sarcoma

**DOI:** 10.3389/fcell.2022.847513

**Published:** 2022-03-01

**Authors:** Zhiyuan Guan, Shengfu Liu, Liying Luo, Zhong Wu, Shan Lu, Zhiqiang Guan, Kun Tao

**Affiliations:** ^1^ Department of Orthopedics, The Shanghai Tenth People’s Hospital of Tongji University, Shanghai, China; ^2^ Nanjing Medical University, Nanjing, China; ^3^ The Department of Ophthalmology, Tongren Hospital, Shanghai Jiao Tong University School of Medicine, Shanghai, China; ^4^ Department of Nursing, Xuzhou Municipal Hospital Affiliated with Xuzhou Medical University, Jiangsu, China; ^5^ Department of Dermatology, Xuzhou Municipal Hospital Affiliated with Xuzhou Medical University, Xuzhou, Jiangsu, China

**Keywords:** ferroptosis, sarcoma, prognosis, tumor microenvironment, bioinformatics

## Abstract

Sarcomas are seen as mixed-up nature with genetic and transcriptional heterogeneity and poor prognosis. Although the genes involved in ferroptosis are still unclear, iron loss is considered to be the core of glioblastoma, tumor progression, and tumor microenvironment. Here, we developed and tested the prognosis of SARC, which is a genetic marker associated with iron residues. The ferroptosis-related gene expression, one-way Cox analysis, and least-selection absolute regression algorithm (LASSO) are used to track prognostic-related genes and create risk assessment models. Finally, immune system infiltration and immune control point analysis are used to study the characteristics of the tumor microenvironment related to risk assessment. Moreover, LncRNA–miRNA–mRNA network was contributed in our studies. We determined the biomarker characteristics associated with iron degradation in gene 32 and developed a risk assessment model. ROC analysis showed that its model was accurately predicted, with 1, 2, 3, 4, and 5 years of overall survival in TCGA cohort of SARC patients. A comparative analysis of settings found that overall survival (OS) was lower in the high-risk than that in the low-risk group. The nomogram survival prediction model also helped to predict the OS of SARC patients. The nomogram survival prediction model has strong predictive power for the overall survival of SARC patients in TCGA dataset. GSEA analysis shows that high-risk groups are rich in inflammation, cancer-related symptoms, and pathological processes. High risk is related to immune cell infiltration and immune checkpoint. Our prediction model is based on SARC ferritin-related genes, which may support SARC prediction and provide potential attack points.

## Introduction

SARC (sarcoma) is a type of stromal cancer with more than 100 subtypes. Sarcomas occur in all age groups and are more general in teens and young adults than in the elderly (Reed et al., 2019). SARC also includes many malignant tumors with different symptoms, behaviors, and results. For example, chemotherapy has only a known effect on a few sarcomas. Although confirmed sarcoma can be removed by surgery or radiotherapy, the incidence of metastatic sarcoma within 5 years is still as high as 50% (Brennan et al., 2014). Only 5% of metastatic patients have a 5-year survival period (Zhu et al., 2020).

Although some of the diagnostic markers of SARC can effectively predict the progress of SARC, they are still at the molecular stage and not yet used in clinical practice. Therefore, diagnostic markers related to undiscovered genes are of great significance for the diagnosis and prediction-related analysis of SARC.

Ferroptosis, an iron-dependent form of programmed cell death, which is linked to pathophysiological conditions. Sensitivity to lipid peroxidation is attributed to many metabolic pathways, such as fatty acid metabolism, iron processing, methionine metabolism, and mitochondrial respiration (Zheng and Conrad, 2020; Jiang et al., 2021). Notably, ferroptosis is related to stroke, ischemic heart disease, liver and kidney damage (Benjamin et al., 2017). In particular, there is strong evidence that high levels of ferroptosis play an important role in the suppression of tumor growth, and the activation of iron levels will enhance the therapeutic effect of anticancer drugs (Li and Li, 2020). Cancer cells store large amounts of iron and active oxygen to stimulate metabolism and growth(Battaglia et al., 2020). Gene dysregulation involved in iron nucleocytosis may promote cancer cell proliferation, invasion, and metastasis (Jung et al., 2019). In addition, CD8 + T cells cause iron loss by controlling the link between cancer cell death mechanism and immune system activation, thereby inhibiting tumor growth (Tang et al., 2020).

In the past, there was an association between many subtypes of sarcoma and apoptosis-related genes such as GPX4, which induces iron loss by increasing MDA, ROS, and intracellular iron levels in osteosarcoma cells (Lin H et al., 2021). LOX promotes tumor deposition and catalyzes the production of lipid hydrogen peroxide in the plasma membrane. In the process of ptosis, ALOX15 protein is always present in the cell membrane (Shintoku et al., 2017). Pharmacological lipid peroxidation inhibitors (such as ferrostatin-1 and lipid peroxidin-1), reactive oxygen species scavengers (such as alpha-tocopherol and glutathione), and iron chelator deferoxamine can inhibit the accumulation of reactive oxygen species; lipid peroxidation oxidizes and heals swamps (Dächert et al., 2020). However, the side effects of ferroptosis limit their applications in treating sarcomas (Blatt, 1994). Therefore, sarcoma iron gout needs more understanding.

These studies focused on genes related to iron-related diseases. We perform extensive bioinformatics analyses based on The Cancer Genome Atlas (TCGA) clinical data to analyze the gene expression levels, DNA methylation, and copy number transfer models. The SARC risk assessment system in TCGA dataset was developed and validated by detecting regulated iron-related genes. In addition, the function and gene clusters were performed to identify possible pathways and mechanisms of ferroptosis metabolism. Our results indicate that four genetic markers can be used as independent predictors of OS in sarcoma patients.

## Materials and Methods

### Analysis of the Ferroptosis-Related Genes in SARC

Ferroptosis-related genes are obtained from the genset (the Molecular Feature Database (MSigDB) version 7.1) (Subramanian et al., 2005; Liberzon et al., 2015), including the 15 gene sets (Mou et al., 2020; Zhang S et al., 2020). After removing overlapping genes, we got a set of genes related to ferroptosis-related gene (FRG), including 32 genes.

### Datasets and Data Processing

As of 30 November 2021, we have obtained data on RNA sequences and summary of 260 SARC patients’ clinical characteristics from TCGA database. The RNA sequence data of 88 normal human ovarian samples were downloaded from the GTEx database. The RNA-seq data and clinical information from the external validation cohort were extracted from the GEO database (GSE21050).

A total of 32 FRGs were obtained from previous studies (Lin W et al., 2021; Ye et al., 2021). The “limma” packages demonstrate the difference between FRG expression in SARC and normal tissues. Then, we contribute 32 protein–protein interaction networks (PPI) to search for interaction finder (STRING).

### Gene Mutation, Methylation, and Copy Number Variation

The “maftool” package is used to create single-nucleotide variation, copy number variation, and 32 FRG cascade plots for SARC patients. GSCA Lite is used to keep methylation alive and analyze the changes in copy number.

### Gene Functional Enrichment Analysis

Genetic ontology (GO), which includes biological process (BP), cell composition (CC), and molecular function classification (MF), uses the “ggplot2” software package in R. In the same way, Kyoto Encyclopedia and Genome uses this software package to analyze (KEGG) genes. A hypergeometric distribution test was applied to detect enrichment terms, and *p* values were adjusted by the false discovery rate (FDR) method with a cutoff FDR <0.05.

### Development of Ferroptosis-Related Prognostic (FRG) Model

Cox regression analysis (COX analysis) is used to investigate the effects of FRGs on prognosis of SARC. The Kaplan–Mayer curve uses the logarithmic test and one-way regression of Cox proportional hazards to generate p (HR) values with 95% confidence intervals (CIs). PRGs with important prognostic value were selected in further analysis. Based on these prognostic-related PRGs, LASSO–Cox regression analysis was used to contribute a prognostic model. According to the average risk score, TCGA–SARC patients were divided into low-risk and high-risk subgroups, and Kaplan–Meier analysis was used to compare the overall survival rates of the two subgroups. Time receiver performance (ROC) analysis is used to evaluate the prediction accuracy and risk assessment of each gene. Taking into account the clinical characteristics, we developed a nomosis prediction schedule to predict the overall survival rate at 1, 3, and 5 years. A forest was used to show the *p*-value, HR, and 95% CI of each variable through the “forest plot” R package.

To evaluate whether the risk score system can serve as an independent predictive index, univariate and multivariate Cox regression analyses were performed with clinicopathologic parameters. All independent prognostic parameters were used to construct a nomogram to predict the 1-, 3- and 5-year OS probabilities by the “rms” package. The concordance index (C-index), calibration, and ROC analyses were used to evaluate the discriminative ability of the nomogram (Alba et al., 2017).

### Immune Related Analysis in Ferroptosis-Related Prognostic (FRG) Model

We used the tumor immune assessment function to analyze the relationship between prognostic FRGs and immune system infiltration, and comprehensively analyze the immune cells infiltrating the portal. The TIM gene module can monitor the relation between the expression of SARC gene and the degree of immune infiltration. Spearman correlation analysis was used to calculate the correlation between gene expression, and the results of tumor mutation burden (TMB) and microsatellite instability (MSI). *p* values less than 0.05 are considered statistically significant.

### Endogenous RNA Network Analysis

To elucidate the possible role of FRGs in SARC, a competitive endogenous RNA (ceRNA) network.miRTarBase (http://mirtarbase.cuhk.edu.cn/) and TarBase V.8 (https://carolina.imis.athenainnovation.gr/diana_tools/web/index.php?r=tarbasev8%2Findex) were contributed and utilized to predict the possible miRNA targets. According to the identified miRNA, it is assumed that StarBase (http://starbase.sysu.edu.cn/) is LncBase v.2 (https://carolina.imis.athena-innovation.gr/diana_tools/web/index.php?r = lncbasev2/index-predicted) is used to predict the interaction between lncRNA and miRNA. In all analyses, *p*< 0.05 was considered to be statistically significant.

### GSEA

The aforementioned software package R was used to calculate the DEG training package. Then GSEA (http://software.broadinstitute.org/gsea/index.jsp) was used to identify the characteristics of high- and low-risk groups.

### TIMER Database Analysis

The result of the infiltration estimation generated by the TIMER algorithm consists of 6 specific subgroups of immune cells, including B cells, CD4 + T cells, CD8 + T cells, macrophages, neutrophils, and dendritic cells (Sturm et al., 2019; Li et al., 2020). We extracted the results of the penetration assessment and evaluated the different results of the penetration assessment of immune cell subsets between high-risk and low-risk populations (Newman et al., 2015).

### Statistical Analysis

All statistical analyses in this study were performed using software R (version 3.6.3) and GraphPad Prism (version 8.0.2). Kaplan–Mayer survival analysis uses the logarithmic series test. Risk factors (HR) and 95% confidence intervals (CI) should be provided when relevant. Both groups were compared with Student’s t-test and the Kruskal–Wallis test. A two-tailed *p* value less than 0.05 was considered statistically significant without specific annotation.

## Results

### Expression of FRGs in SARC

**FIGURE 1:**
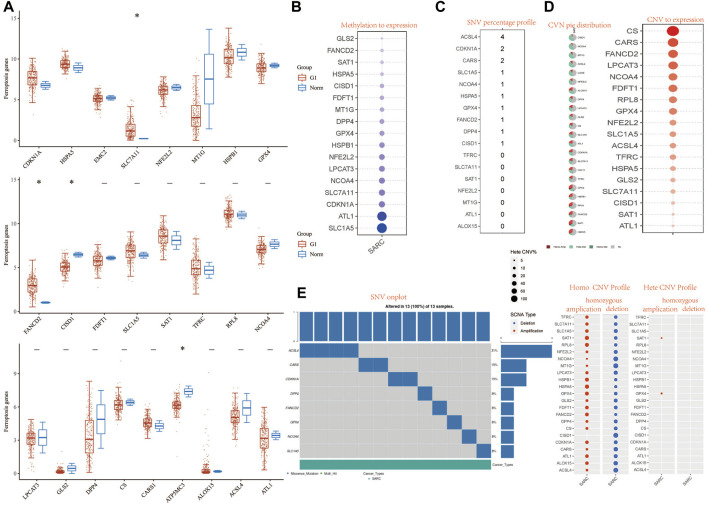
Landscape of genetic and expression variation of FRG in SARC. **(A)** The expression of 32 FRG in SARC, Tumor, blue; normal, red. The upper and lower ends of the boxes represent the interquartile range of values. The lines in the boxes represent median value. **(B)** Methylation to expression. **(C)** SNV percentage profile. **(D)** CNV pie distribution and CNV to expression of 32 FRG in the SARC cohort. **(E)** SNV onplot, homo CNV profile, and hete CNV profile of 32 FRG in the SARC cohort. ***p< 0.001. FRG, ferroptosis-related gene; SARC, sarcoma; SNP, single nucleotide polymorphism; INS, insertion; DEL, deletion; SNV, single-nucleotide variation; CNV, copy number variation.

First, we use TCGA data to study the expression of 32 FRGs in SARC and normal tissues. The total amount of SARC is 4 FRGs. For example, the expression of SLC7A11, FANCD2, and CISD1 was higher in tumor tissues than that in normal tissues, but ATP5MC3 was lower. A GeneMANIA network showed that FANCD2, SLC7A11, CISD1, and ATP5MC3 were performed to detect these FRG interactions. The minimum required interaction score is 0.9. The heat map of results showed that FANCD2, SLC7A11, CISD1, and ATP5MC3 are key genes (Figure 1, SupplementaryFigure S1, Supplementary Table S1).

### Landscape of Genetic Variation, Methylation, and Copy Number Variation

We first analyzed the relationship between FRG and methylation. In all FRGs, we found that the methylation rate of SLC1A5 and ATL1 is associated with a methylation level (Figure 1B). Then we analyzed the percentage plot of individual nucleotide variation on the chart. The ACSL4 mutation frequency is high (Figure 1C). We summarized the copy number and frequency of 32 FRG somatic mutations in SARC. As shown in Figures 1D,E, we found that CISD1 and NCOA4 have a higher level of CNV pie distribution, and CS, CARS, FANCD2, and LPCAT3 have a higher CNV expression level. ALOX15 has a high circulating distribution of CVN (copy number change), while CISD1, ACSL4, and ALOX15 show high levels of homozygous SARC deletion. Supplementary Figure S5C shows that TP53 and RB1 have higher levels of genomic alteration. The mutation count, TMB, fraction genome altered, aneuploidy score, MSIsensor score, radiation therapy, and Onc Tree code, and cancer type details also show significant difference in genome changes of FRGs (Supplementary Table S3).

### Functional Enrichment Analysis

**FIGURE 2:**
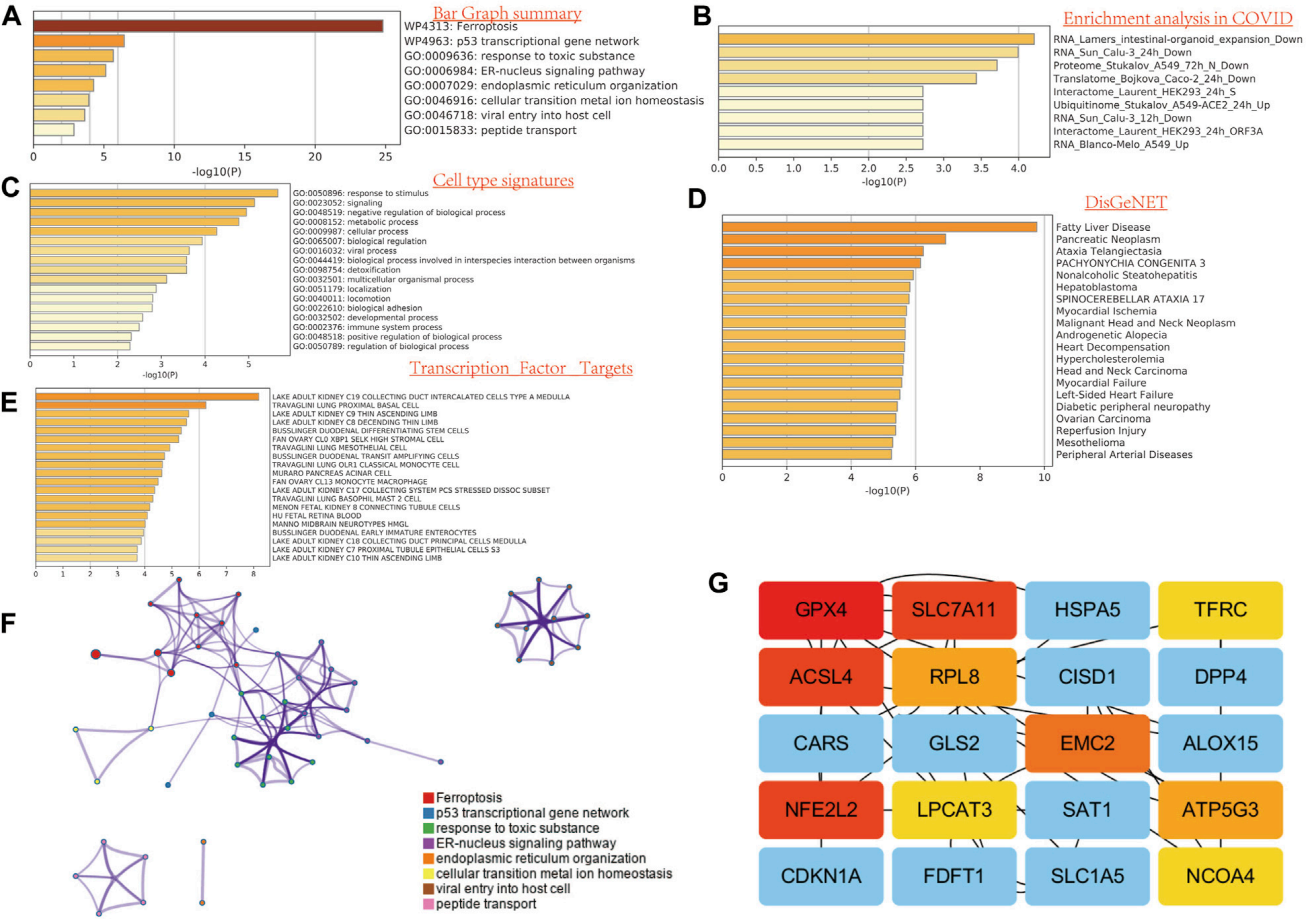
Functional enrichment analysis of FRG in SARC. **(A)** Bar graph summary. **(B)** Enrichment analysis in COVID. **(C)** Cell type signature. **(D)** DisGeNET. E, transcription factor targets. **(E)** GO enrichment analysis. **(F)** Network analysis. **(G)** Network analysis between top 20 genes.

To illustrate the role of FRGs, we uses GO and KEGG databases to analyze the FRGs pathway. We found that these 32 FRGs are mainly involved in the p53 transcriptional gene network, response to toxic substance, ER–nucleus signaling pathway, endoplasmic reticulum organization, cellular transition metal ion homeostasis, and peptide transport are involved in the process of ferroptosis in GO assay (Figure 2A). For COVID related GO enrichment analysis, RNA lamers intestinal organoid expansion, RNA Sun calu-3 24 h, proteome stukalov, translatome luarant also play an important role in the ferroptosis related pathway. The cell type signature for example response to stimulus, signaling and negative regulation of biological process and transcription factor target such as lake adult kidney c19 collecting duct intercalated cells types a medulla also play potential role in ferroptisis related pathway. In addition, the analysis of the KEGG pathway showed that the 32 FRGs are mainly involved in the TOR, RTX and EMT (Figure 2A). We also compiled the percentage of the global channel, the percentage of the heat map, and the connection network in Supplementary Figure S4 (Supplementary Tables S3–4).

### Ferroptosis-Related Prognostic Gene Model

**FIGURE 3:**
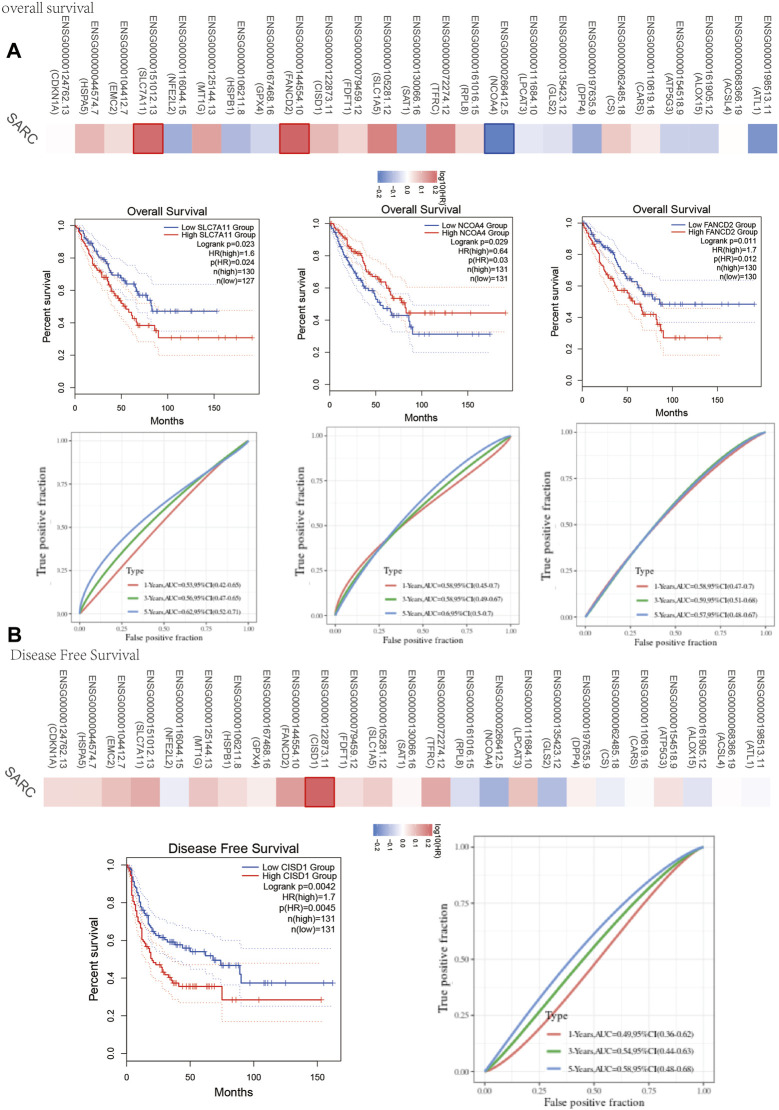
Prognosis value of FRG in SARC. **(A)** The overall survival curve of SLC7A11, NCOA4, and FANCD2 in SARC patients in the high-/low-expression group. **(B)** Disease free survival curve of CISD1 in SARC patients in the high-/low-expression group. FRG, ferroptosis-related gene; SARC, sarcoma.

**FIGURE 4:**
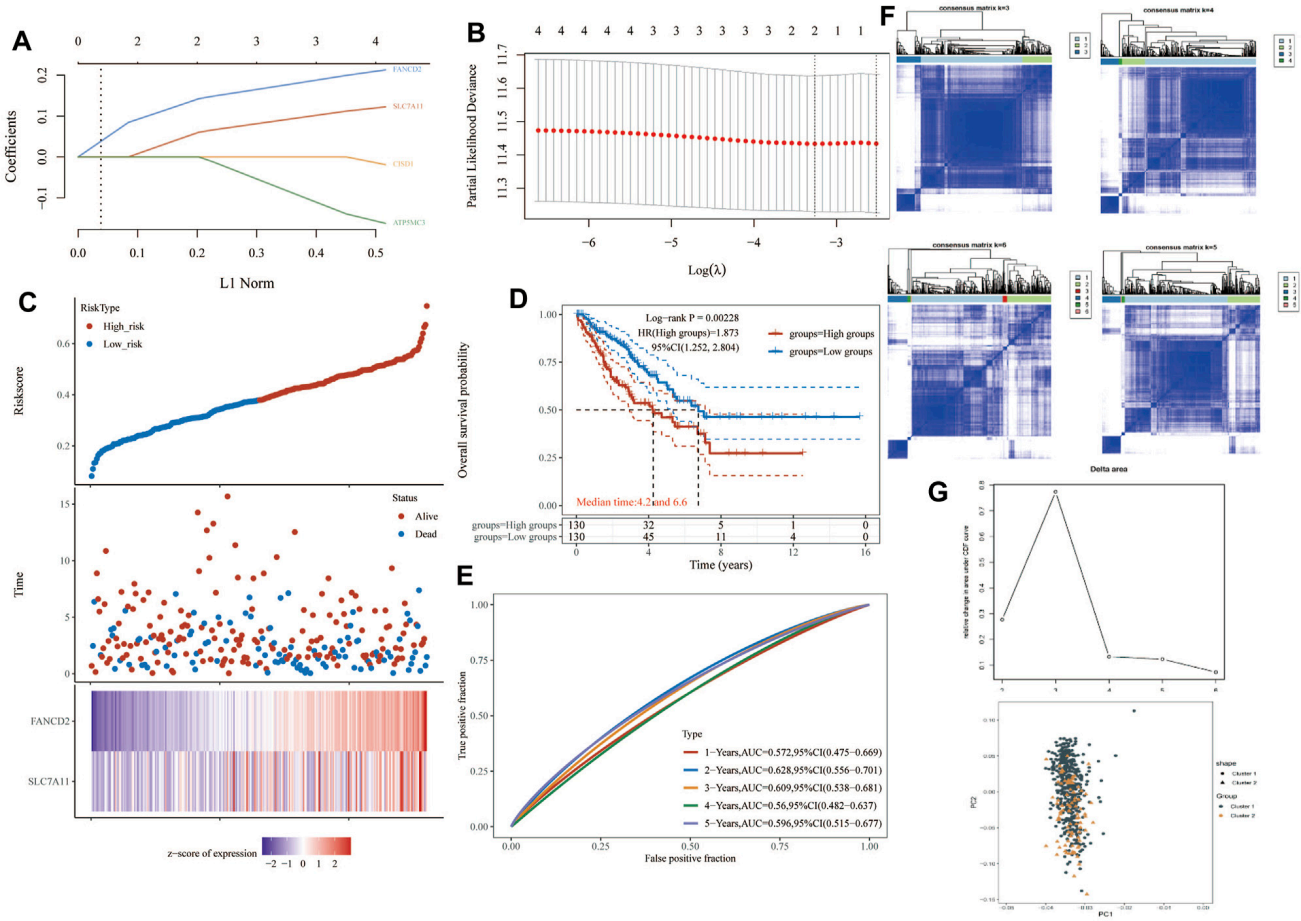
Construction of a prognostic FRG model. **(A)** LASSO coefficient profiles of the four PRGs. **(B)** Plots of the ten-fold cross-validation error rates. **(C)** Distribution of risk score, survival status, and the expression of four prognostic PRGs in SARC. **(D,E)** Overall survival curves for SARC patients in the high-/low-risk group and the ROC curve of measuring the predictive value. **(F,G)** Prognostic FRG model. FRG, ferroptosis-related gene; SARC, sarcoma.

One-way Cox regression analysis was used to select the prognostic FRGs for the genetic prognostic model. As a result, a total of 4 genes with prognostic values were identified. Kaplan–Mayer survival curves are shown in Figures 3A,B. For overall survival, we found that the high expression of SLC7A11(*p* = 0.023) and FANCD2 (*p* = 0.011) was correlated with good prognosis, but that of NCOA4 was linked with poor prognosis. For disease-free survival, we found that high expression of CISD1 (*p* = 0.0042) links with better prognosis (Figure 2C). Based on these 4 prognostic FRGs, LASSO–Cox regression analysis was performed to construct a prognostic gene model (Figure 4). According to risk assessment, SARC patients are divided into two groups. The risk results, survival status, and expression distribution of these genes are shown in Figure 4. The higher the risk score, the higher the risk of death and the shorter the survival time (Figure 4C). The Kaplan–Meier curve shows that the overall survival rate of high-risk patients is not only higher than that of low-risk patients (average time = 4.2 and 6.6 years, *p* = 0.000228, Figure 4D) but also 1 year, 2, 3, 4 year annual, and 5-year ROC curves are 0.572, 0.628, 0.609, 0.56, and 0.596, respectively.

### Building a Predictive Nomogram

**FIGURE 5:**
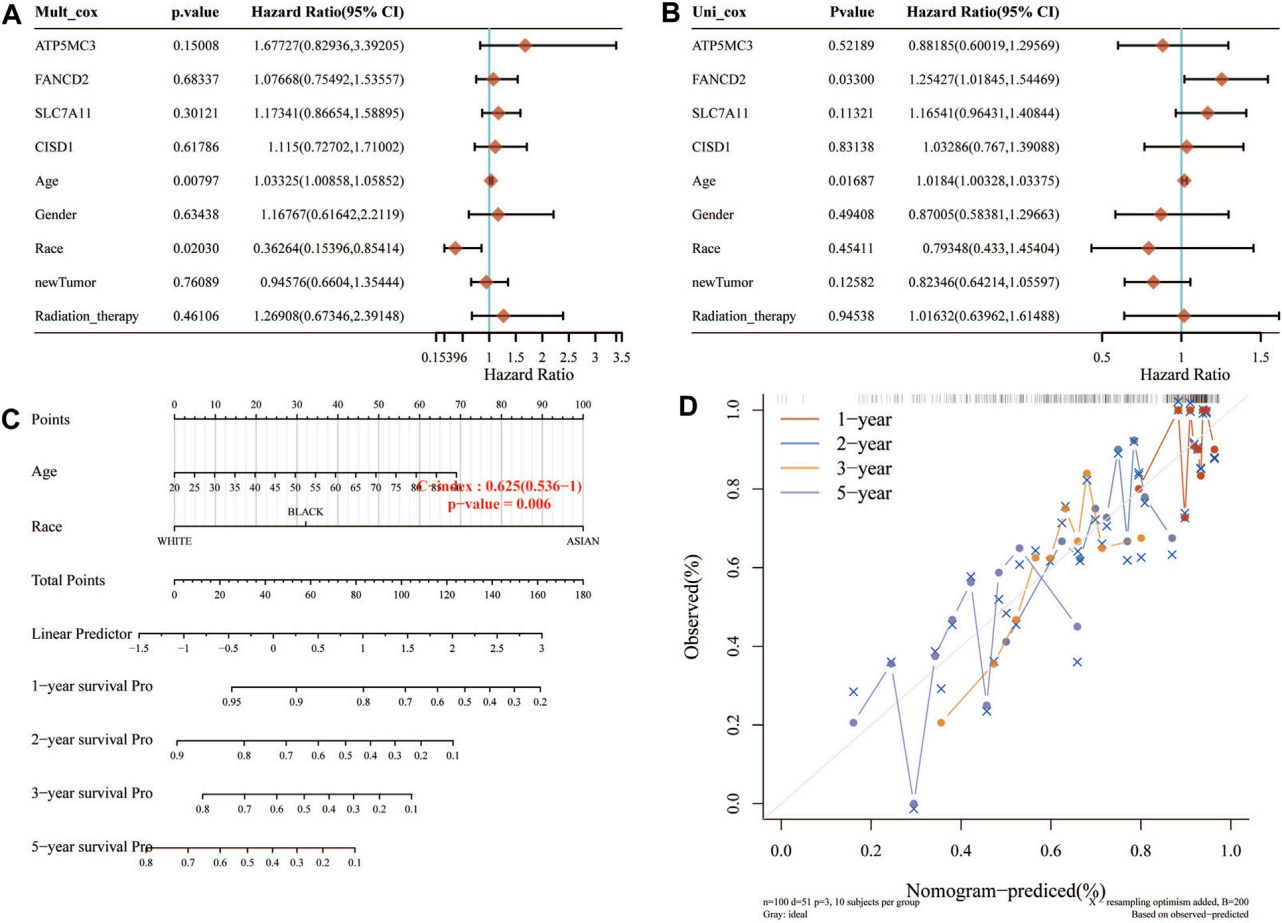
Construction of a predictive nomogram. **(A,B)** Hazard ratio and *p*‐value of the constituents involved in univariate and multivariate Cox regression analyses considering clinical the parameters and four prognostic FRG in SARC. **(C,D)** Nomogram to predict the 1-, 2-, 3-, and 5-year overall survival rate of SARC patients. Calibration curve for the overall survival nomogram model in the discovery group. A dashed diagonal line represents the ideal nomogram. FRG, ferroptosis-related gene; SARC, sarcoma.

Considering the clinicopathologic and prognostic characteristics of FRGs, we also compiled the prognostic nomogram of the survival prediction table. One-dimensional and multi-dimensional analyses showed that NOD1 and pT staging, pN staging, and pM staging are independent factors affecting the prognosis of SARC patients (Figure 5). The chart with predictable names shows that the 1, 2, 3, and 5-year overall survival of the entire cohort can be better predicted than the ideal model (index C: 0.625 (0.536–1), *p*< 0.01).

### Immune Infiltration and Immune Survival in SARC

**FIGURE 6:**
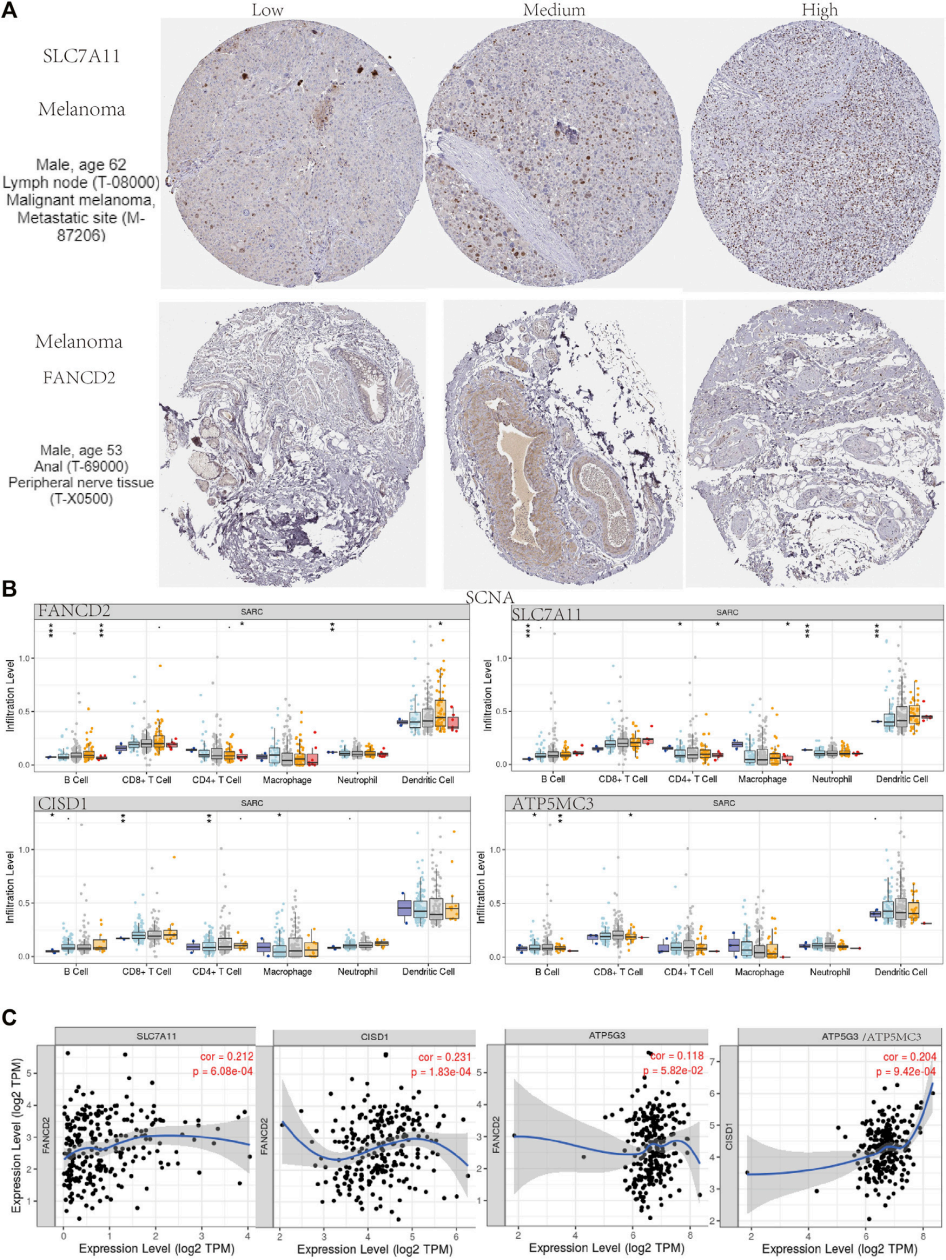
Human Protein Atlas immunohistochemical analysis of SARC. **(A)** SCNA of SARC in FANCD2, SLC7A11, CISD1, and ATP5MC3. **(B)** Correlation analysis among FANCD2, SLC7A11, CISD1, and ATP5MC3 **(C)**.

**FIGURE 7:**
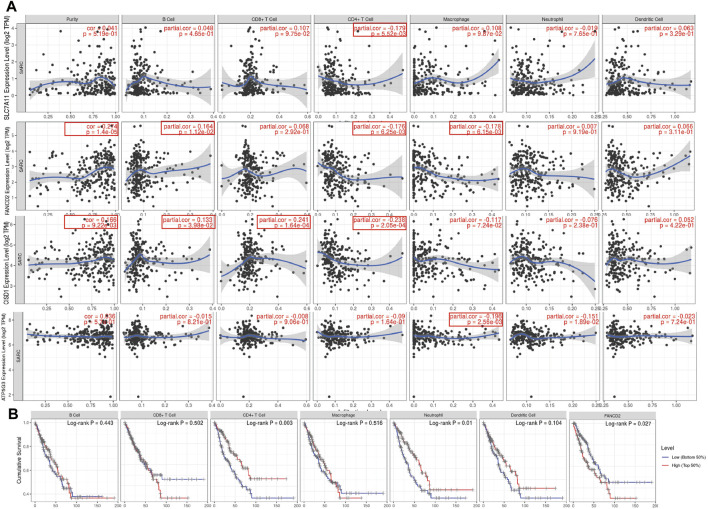
Association between seven prognostic PRG and immune infiltration (TIMER). **(A)** The association between the abundance of immune cells and the expression of FANCD2, SLC7A11, CISD1, and ATP5MC3 in SARC. FRG, ferroptosis-related gene; SARC, sarcoma. **(B)** Cumulative survival of B cell, CD8+ T cell, CD4+ T cell, marcrophage, neutrophil, and dendritic cell of FRGs in SARC.

Immunohistochemical results suggested SLC7A11 and FANCD2 staining in melanoma (Figure 6A). Ferroptosis plays an important role in the creation of tumor immune microenvironment. In our study, we also used the TIMER database to clarify the relationship between the expression of prognostic FRG (FANCD2, SLC7A11, CISD1, and ATP5MC3) and SARC immune filtration and somatic copy number variation. For example, B cells in FANCD2 are significantly expanded, lacking CD4^+^ T cells and macrophage branching. However, FANCD2 have no significant difference in SLC7A11, CISD1, and ATP5MC3 (Figure 6). SLC7A11 was negatively correlated with CD4+ T cells, and FAND2 was negatively correlated with CD4+ T cells and macrophages. The data show that FANCD2 and CISD1 are positively correlated with B cells. CISD1 was also positively correlated with CD8+ T cells but negatively correlated with CD4+ T cells. ATP5G3 was negatively correlated with macrophages (Figure 7A). Immune survival (Figure 7B) shows that lower CD4 + T cells and neutrophils level also have better prognosis. Moreover, a lower FANCD2 level also predicted poor prognosis.

### Drug Sensitivity, TMB, and MSI in SARC

**FIGURE 8:**
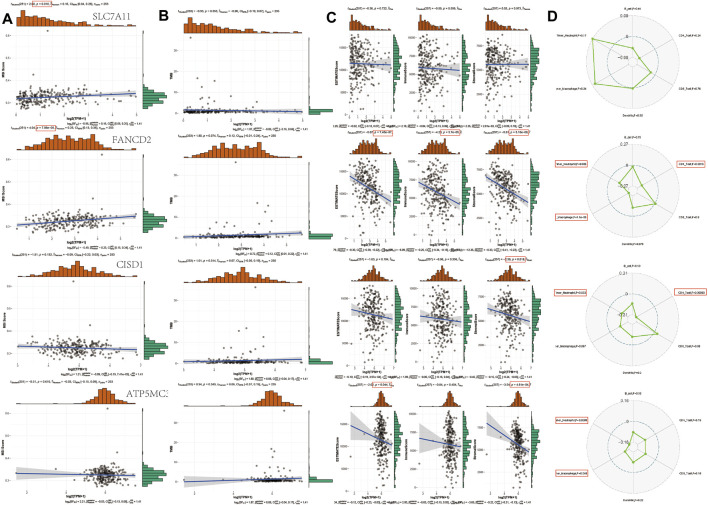
TMB, MSI, and ESTIMATE Score analysis of FRG in SARC. **(A)** Correlation between 4 prognostic FRGs and MSI in SARC. **(B)** Correlation between 4 prognostic FRGs and TMB in SARC. **(C)** Correlation between 4 prognostic FRGs and ESTIMATE Score in SARC. **(D)** Correlation between 4 prognostic FRGs and immune score in SARC. TMB, tumor mutation burden; MSI, microsatellite instability; LUAD, lung adenocarcinoma; FRG, ferroptosis-related gene; CTRP, Cancer Therapeutics Response Portal; GDSC, Genomics of Drug Sensitivity in Cancer.

The instability of TMB and microsatellites can be used as biomarkers to predict the effect of tumor immunotherapy (Campbell et al., 2017; Latham et al., 2019; Samstein et al., 2019; Chan et al., 2020). The aforementioned analysis shows that FRGs are negatively correlated with tumor immune system infiltration. To determine whether this FRG can also be used as a biomarker for drug screening, we analyzed the correlation between FRG and TMB and MSI in SARC. The results show that MSI is positively correlated with SLC7A11 (*p* = 0.010) and FANCD2 (*p* = 7.06E-5). In addition, the TMB also negatively correlated with FANCD1, SLC7A11, CISD1, and ATP5MC3. SLC7A11 has negatively correlated with ESTIMATE score (*p* = 7.45E-7), immune score (*p* = 3.7E-5), and Stromal Score (*p* = 5.16E-8). FANCD2 was negatively correlated with Stromal Score (*p* = 0.018). CISD1 negatively correlated with ESTIMATE score (*p* = 0.044) and Stromal Score (*p* = 4.81E-4). Immune score shows that FANCD2 was correlated with neutrophils, macrophages, and CD4+ T cells. CISD1 were correlated with neutrophils and CD4^+^ T cells. ATP5MC3 correlated with neutrophils and macrophages (Figure 8). The immune checkpoint and immune pathway are shown in Supplementary Figure S2. Analyzing the correlation between gene expression and available drugs is key to setting treatment goals. In our study, we analyzed 14 genes that are significantly related to some or most anticancer drugs, such as FANCD2, HSPA5, NFE2L2, ACSL4, and DPP4 in the Cancer Therapeutic Response Portal Database (Supplementary Figure S2).

### Contribute an miRNA–LncRNA–mRNA Network

To elucidate the molecular mechanism of FRGs in SARC, we constructed an mRNA–miRNA–lncRNA interaction network. We first investigate the correlation hub gene, and Ven plot found that 101 hub genes have been analyzed, as shown in Supplementary Figure S3A and Table 1. The ferroptosis-related gene pathway analysis found the energy produced by the oxidation of organic compounds and cellular respiration in the BP pathway and mitochondrial inner membrane, mitochondrial protein complex in CC pathway, electron transfer activity, NADH dehydrogenase activity in MF and Parkinson disease, oxidative phosphorylation, and non-alcoholic fatty liver disease in KEGG (Supplementary Figure S3A,B).

**TABLE 1:** Hub gene analysis by GO analysis.

node_name	MCC	DMNC	MNC	Degree	EPC	Bottleneck	Eccentricity	Closeness	Radiality	Betweenness	Stress	Clustering coefficient
SAT1	2	0	1	4	13.791	1	0.16667	8.05	5	2	24	0
HSPA5	3	0.61557	2	6	14.376	3	0.2	9.73333	5.66667	76	2,544	0.13333
SLC1A5	2	0.61557	2	4	12.805	1	0.16667	7.58333	4.80952	0	0	0.33333
GLS2	2	0.61557	2	4	13.12	1	0.16667	7.58333	4.80952	0	0	0.33333
FDFT1	1	0	1	2	9.216	1	0.14286	6.17619	4.04762	0	0	0
TFRC	3	0	1	6	12.628	4	0.2	9.28333	5.57143	96	3,016	0
DPP4	1	0	1	2	9.464	1	0.16667	6.58333	4.61905	0	0	0
HSPB1	2	0	1	4	11.097	2	0.16667	7.46667	4.80952	40	1704	0
CS	1	0	1	2	8.169	1	0.14286	5.66905	3.85714	0	0	0
NCOA4	3	0	1	6	13.921	22	0.25	10.58333	6.09524	212	6,504	0
NFE2L2	6	0.61795	3	10	15.15	3	0.2	10.9	5.80952	71.66667	1776	0.08889
CDKN1A	1	0	1	2	11.024	1	0.16667	7.21667	4.85714	0	0	0
CARS	1	0	1	2	9.431	1	0.14286	6.25952	4.09524	0	0	0
CISD1	3	0.61557	2	6	13	4	0.2	9.11667	5.52381	84	2,912	0.13333
RPL8	4	0.61557	2	8	12.874	2	0.16667	9	5.04762	52	1,640	0.07143
EMC2	5	0.61795	3	8	12.739	2	0.16667	8.83333	5	42	1,568	0.14286
ATP5G3	4	0.61795	3	6	12.671	1	0.16667	8.33333	4.95238	2	24	0.26667
GPX4	11	0.47549	6	14	15.35	22	0.25	12.66667	6.28571	259.3333	7,152	0.10989
ALOX15	2	0.61557	2	4	14.017	1	0.2	8.48333	5.38095	0	0	0.33333
SLC7A11	6	0.61795	3	10	15.039	3	0.2	10.65	5.71429	78	1896	0.13333
LPCAT3	3	0.61557	2	6	14.695	1	0.2	9.15	5.47619	13.33333	640	0.13333
ACSL4	6	0.56839	4	8	15.093	1	0.2	9.98333	5.61905	7.66667	104	0.21429

The data identified 5 miRNAs such as hsa-mir-140-3p, hsa-mir-221-3p, hsa-mir-222-3p, and has-miR-16-5p as the targeting mRNA binding to CISD1, ATP5MC3, SLC7A11, and FANCD2 in ENCORI dataset. The high levels of hsa-mir-29c-3p, hsa-mir-15b-5p, hsa-mir-425-5p, and hsa-mir-424-5p were correlated with poor prognosis in SARC (Supplementary Figure S6 and Table 2 and 3). Based on this result, we also checked its upstream lncRNA target to construct an miRNA–lncRNA axis. As shown in Supplementary Figure S4, we found the miRNA–LncRNA network in has-mir-29c-3p with 11 lncRNA in Supplementary Figure S4A. We also analyzed the expression level of SARC in Supplementary Figure S4B. The higher level of 3 lncRNA such as TPL1P1 (*p* = 0.0017), HSP90AB3P (*p* = 0.00044), and GAPDHP (*p* = 0.0068) were correlated with poor prognosis (Supplementary Figure S4). According to mirTarBase and TarBase V.8, Has-mir-29c-3p has been identified as the target of the ceRNA network (Supplementary Figure S6). We found that 52 common lncRNAs have been found in mirTarBase and TarBase V.8., and the LncRNA–miRNA–mRNA network has also been contributed in SARC (Supplementary Figure S6).

**TABLE 2:** Prediction of miRNA binding to ATP5MC3.

Gene name	miRNA name	PITA	RNA22	miRmap	microT	miRanda	PicTar	TargetScan	Number
ATP5MC3	hsa-miR-425-5p	1	0	1	1	1	1	1	6
ATP5MC3	hsa-miR-15a-5p	1	0	1	1	1	0	0	4
ATP5MC3	hsa-miR-16-5p	1	0	1	1	1	0	0	4
ATP5MC3	hsa-miR-27a-3p	1	0	1	0	1	0	1	4
ATP5MC3	hsa-miR-29a-3p	1	0	1	0	1	0	1	4
ATP5MC3	hsa-miR-29b-3p	1	0	1	0	1	0	1	4
ATP5MC3	hsa-miR-139-5p	1	0	1	0	1	0	1	4
ATP5MC3	hsa-miR-15b-5p	1	0	1	1	1	0	0	4
ATP5MC3	hsa-miR-27b-3p	1	0	1	0	1	0	1	4
ATP5MC3	hsa-miR-195-5p	1	0	1	1	1	0	0	4
ATP5MC3	hsa-miR-29c-3p	1	0	1	0	1	0	1	4
ATP5MC3	hsa-miR-424-5p	1	0	1	1	1	0	0	4
ATP5MC3	hsa-miR-425-5p	1	0	1	1	1	0	0	4
CISD1	hsa-miR-140-3p	1	1	0	1	0	0	1	4
FANCD2	hsa-miR-221-3p	1	0	1	0	1	1	0	4
FANCD2	hsa-miR-222-3p	1	0	1	0	1	1	0	4
SLC7A11	hsa-miR-26a-5p	1	0	1	1	1	1	1	6
SLC7A11	hsa-miR-26b-5p	1	0	1	1	1	1	1	6
SLC7A11	hsa-miR-27a-3p	1	0	1	1	1	1	1	6
SLC7A11	hsa-miR-27b-3p	1	0	1	1	1	1	1	6
SLC7A11	hsa-miR-329-3p	2	0	2	2	0	0	0	6
SLC7A11	hsa-miR-362-3p	2	0	2	2	0	0	0	6
SLC7A11	hsa-miR-1297	1	0	1	1	1	1	1	6

**TABLE 3:** Prognostic values of potential upstream miRNAs of in TCGA sarcoma cancer cohort.

miRNA	Cancer	Cox coefficient	*p*-value	FDR Corrected	Rank	Median Expression	Mean Expression
hsa-miR-29c-3p	ATP5MC3	−0.356	0.0011	0.0125	40	273.42	444.98
hsa-miR-15b-5p	ATP5MC3	0.262	0.014	0.0625	97	196.27	270.8
hsa-miR-425-5p	ATP5MC3	0.233	3.30E-02	9.88E-02	152	83.61	111.13
hsa-miR-424-5p	ATP5MC3	0.191	0.046	0.12	174	145.56	238.01
hsa-miR-362-3p	SLC7A11	0.19	0.062	0.144	196	2.62	4.12
hsa-miR-26a-5p	SLC7A11	0.171	0.11	0.212	234	1,544.81	4,683.31
hsa-miR-16-5p	ATP5MC3	0.149	1.20E-01	2.21E-01	238	395.86	429.71
hsa-miR-29a-3p	ATP5MC3	−0.127	0.23	0.355	295	2,448.34	3,355.48
hsa-miR-195-5p	ATP5MC3	−0.115	0.24	0.365	299	31.24	42.44
hsa-miR-222-3p	FANCD2	0.112	2.90E-01	4.16E-01	316	72.38	117.04
hsa-miR-26b-5p	SLC7A11	0.1	0.33	0.461	324	334.61	427.63
hsa-miR-221-3p	FANCD2	0.095	3.70E-01	5.00E-01	335	280.69	469.1
hsa-miR-140-3p	CISD1	0.071	0.46	0.586	354	1,235.76	1,534.06
hsa-miR-27b-3p	ATP5MC3	−0.063	0.56	0.669	381	895.4	1,123.92
hsa-miR-27b-3p	SLC7A11	−0.063	0.56	0.669	381	895.4	1,123.92
hsa-miR-27a-3p	ATP5MC3	0.059	0.58	0.685	383	836.55	992.59
hsa-miR-27a-3p	SLC7A11	0.059	0.58	0.685	383	836.55	992.59
hsa-miR-29b-3p	ATP5MC3	−0.024	0.82	0.872	427	223.34	393.42
hsa-miR-139-5p	ATP5MC3	0.003	0.98	0.984	449	41.27	61.09
hsa-miR-15a-5p	ATP5MC3	−0.001	9.90E-01	9.92E-01	454	201.56	223.4

## Discussion

Ferroptosis is a recently found form of cell-regulated death related to the metabolism pathway in human health (Liang et al., 2019). Accumulation of iron (II) and lipid peroxidation play important roles in causing ferroptosis. Iron chelators and lipophilic antioxidants can inhibit ferroptosis (Su et al., 2020). However, there is increasing evidence that iron poisoning plays an important role in neuro-diseases, including neurodegenerative diseases and cardiovascular diseases (Zhou et al., 2020). In addition, a thorough FRG landscape analysis of soft tissue sarcoma revealed a new type of FRG related to cancer and prognosis (Huang et al., 2021). Ferroptosis has recently become a treatment target and potential biomarkers for sarcoma (Chen et al., 2021).

We performed this study for the first time using gene information of SARC from open databases. First, we chose 32 genes, which are related to ferroptosis. Among them, four ferroptosis genes were analyzed using Cox and LASSO regression to select potential prognostic markers of one variant and then to construct a prognostic model. Among them, the expression level of two genes (SLC7A11 and FANCD2) was negatively correlated with OS, and the expression level of one gene (NCOA4) was positively correlated with OS. These data are consistent with previous results. Sun et al. found that the inhibition of GSH and SLC7A11 is the main cause of EMT and iron deficiency in A549 cells (Sun et al., 2021). SLC7A11 is becoming a potential new cancer treatment target. This article briefly introduces the structure and function of SLC7A11 (Lin et al., 2020). FANCD2 has played a new role in reducing ferroptosis. FANCD2 can be used as a target for the development of new cancer therapies aimed at reducing the side effects of ferroptosis (Song et al., 2016). ATP5MC3 is better predictor in a risk prognosis model and also be defined as potential drug for treatment of prostate cancer.

We also analyzed the functional enrichment of these FRGs and found that these 32 FRGs are mainly involved in the regulation of the p53 gene transcription network, response to toxic substance, ER–nucleus signaling pathway, endoplasmic reticulum organization, cellular transition metal ion homeostasis, and peptide transport (Kang et al., 2019). It has been confirmed that the level of glutathione in intestinal tissue is the most influential metabolic pathway related to glutathione/GPx 4 after exposure to microcystin (Zhang et al., 2021). Colitis mice is evidently induced by ferroptosis, which is mediated by stress signals from the endoplasmic reticulum (Xu et al., 2020). The enigmatic lipid peroxidation product is believed to be the direct agent of ferritin metabolism, a special death program caused by glutathione peroxidase 4 (GPX4) insufficiency (Kagan et al., 2017).

In recent years, it has fundamentally changed the way cancer is treated (Dupont et al., 2021), which pointed out that by integrating multi-dimensional biological data and clinical characteristics, highly heterogeneous tumors can be classified into more specific subtypes for personalized treatment (Zhang H et al., 2020). In fact, the accumulation of evidence based on molecular profiling has established itself in the subgroup of cancer patients representing different phenotypes, prognosis, and treatment response (Tan et al., 2019). According to the characteristics of immunogen expression, patients with sarcoma can be divided into two subtypes: prognosis and clinical importance. Patients with high-risk immune subtypes are more sensitive to immune checkpoint blockers (Francescutti and Skitzki, 2012; Tsukahara et al., 2016).

In addition, we developed a ferritin-related gene-based nomogram prediction model for predicting OS in SARC patients. The calibration table and ROC analysis show that the nomogram has a reliable predictive ability for the queue TCGA operating system. The Nomap model can be used to determine the patient’s prognosis and make follow-up plans.

GSEA showed that the drug is more sensitive to immune response and tumor progression in the high-risk group. In Kaposi’s sarcoma, chemotherapy with vincristine, bleomycin, and etoposide appears to improve overall survival, survival, and quality of life (Macken et al., 2018). Methotrexate also plays an important role high-grade osteosarcoma in children and young adults (van Dalen et al., 2011; Abe et al., 2021). The overall biological response in Ewing’s sarcoma exposure shows differences in response (Aryee et al., 2013).

miRNA and lncRNA play a role in a variety of biological and pathological processes, such as apoptosis, cell cycle, migration, invasion, regulating proliferation, metastasis, EMT, and resistance to drugs. It plays an important role and has been extensively studied. mRNAs are target through transcription or post-transcription (Wang et al., 2020). Therefore, the LncRNA–miRNA–mRNA network was contributed in our studies. Perilous studies found that LncRNA TTN-AS1 regulates apoptosis and drug resistance in osteosarcoma cells through the miR-134-5p/MBTD1 axis (Fu et al., 2019). Zhang et al. also investigated that LncRNA axis SNHG3/miRNA-151a-3p/RAB22A regulates osteosarcoma invasion and migration (Zheng et al., 2019).

Advances in cancer treatment are increasingly recognizing a more promising approach to ferroptosis in the development of effective combination therapies (Friedmann Angeli et al., 2019). As first-line treatment options for patients with SARC are developed, the biology of the tumor and the tumor microenvironment should be considered in order to obtain optimal benefit from treatment strategies. The expression of NCOA4 may be a potential new factor that promotes the stratification of SARC and/or immunotherapy in patients with iron hook, which may be an important factor in predicting the recurrence of SARC patients. In addition, the modulation of SLC7A11 overexpressed in many types of cancers and is associated with patients’ poor prognosis (Lin et al., 2020). In addition, FANCD2 genes were correlated with the diagnostic and prognostic factors of low-grade glioma and breast cancer (Fagerholm et al., 2013; Liu et al., 2020). CISD1 was also regarded as a potentially effective tool for prognostic of pancreatic cancer (Yang et al., 2022). It means that ferroptosis-related genes show great potential during many cancer therapies (Xu et al., 2019).

The major limitation in this study is the lack of available analysis of data heterogeneity and platform differences based on a large number of normal and tumor samples. Our study shows that the disease-related level and extreme harshness of TNM staging are not independent prediction-related factors for OS in SARC patients. This difference may be due to heterogeneity of the data or different classification and classification models (Warren and Harrison, 2018). In addition, the group cancer type information in TCGA database is mainly limited to sarcoma groups, so it is difficult to extrapolate these results to different types and locations of sarcomas. The development of this field also requires further efforts to verify the results of bioinformatics predictions, including the detection of Western blotting proteins or immunohistochemical staining, to promote the analysis of iron dependence *in vivo* and *in vitro* and immunotherapy functions.

## Conclusion

In general, ferroptosis induction and immunotherapy are now the main advances in the treatment of SARC. With a deeper understanding of bleomycin therapy biology and resistance mechanisms, ferroptosis-based combination therapy has received more and more attention. We first found that the high expression of SLC7A11, FANCD2, CISD1, and ATP3MC3 genes related to ferroptosis, which is related to the signal transduction of the infiltrating immune cell receptor in SARC. Therefore, it is expected that SLC7A11, FANCD2, CISD1, and ATP3MC3 will be used as new markers to identify patients who may undergo adequate ferroptosis induction therapy or combined immunotherapy.

## Data Availability

The original contributions presented in the study are included in the article/Supplementary Material; further inquiries can be directed to the corresponding authors.
