# Erratum: Identification of ferroptosis-related genes as biomarkers for sarcoma

**DOI:** 10.3389/fcell.2022.995199

**Published:** 2022-08-19

**Authors:** Frontiers Production Office

**Affiliations:** Frontiers Media SA, Lausanne, Switzerland

**Keywords:** ferroptosis, sarcoma, prognosis, tumor microenvironment, bioinformatics

Due to a production error, the affiliation of LL was erroneously omitted. The correct author list and affiliation list appear below:

Zhiyuan Guan^1†^*, Shengfu Liu^2†^, Liying Luo^3†^, Zhong Wu^1^, Shan Lu^4^*, Zhiqiang Guan^5^* and Kun Tao^2^*

^1^Department of Orthopedics, The Shanghai Tenth People’s Hospital of Tongji University, Shanghai, China

^2^Nanjing Medical University, Nanjing, China

^3^The Department of Ophthalmology, Tongren Hospital, Shanghai Jiao Tong University School of Medicine, Shanghai, China

^4^Department of Nursing, Xuzhou Municipal Hospital Affiliated with Xuzhou Medical University, Jiangsu, China

^5^Department of Dermatology, Xuzhou Municipal Hospital Affiliated with Xuzhou Medical University, Xuzhou, Jiangsu, China

The publisher apologizes for this mistake. The original version of this article has been updated.

