# Supplementary material for: Identification of Ferroptosis-Related Genes as Biomarkers for Sarcoma
Source: Front Cell Dev Biol. 2022 Mar 1;10:847513. doi: 10.3389/fcell.2022.847513 (PMC8929291; doi:10.3389/fcell.2022.847513)
Supplement: Supplementary file 1 [file DataSheet2.docx]

| Supplementary Table 1. The abbreviations of multiple cancer types, ferroptosis regulators and immune-related checkpoints in this study. | |
| --- | --- |
| Abbreviation | Full name |
| ACC | Adrenocortical carcinoma |
| BLCA | Bladder Urothelial Carcinoma |
| BRCA | Breast invasive carcinoma |
| CESC | Cervical squamous cell carcinoma and endocervical adenocarcinoma |
| CHOL | Cholangiocarcinoma |
| COAD | Colon adenocarcinoma |
| DLBC | Lymphoid Neoplasm Diffuse Large B-cell Lymphoma |
| ESCA | Esophageal carcinoma |
| GBM | Glioblastoma multiforme |
| HNSC | Head and Neck squamous cell carcinoma |
| KICH | Kidney Chromophobe |
| KIRC | Kidney renal clear cell carcinoma |
| KIRP | Kidney renal papillary cell carcinoma |
| LAML | Acute Myeloid Leukemia |
| LGG | Brain Lower Grade Glioma |
| LIHC | Liver hepatocellular carcinoma |
| LUAD | Lung adenocarcinoma |
| LUSC | Lung squamous cell carcinoma |
| MESO | Mesothelioma |
| OV | Ovarian serous cystadenocarcinoma |
| PAAD | Pancreatic adenocarcinoma |
| PCPG | Pheochromocytoma and Paraganglioma |
| PRAD | Prostate adenocarcinoma |
| READ | Rectum adenocarcinoma |
| SARC | Sarcoma |
| SKCM | Skin Cutaneous Melanoma |
| STAD | Stomach adenocarcinoma |
| TGCT | Testicular Germ Cell Tumors |
| THCA | Thyroid carcinoma |
| THYM | Thymoma |
| UCEC | Uterine Corpus Endometrial Carcinoma |
| UCS | Uterine Carcinosarcoma |
| UVM | Uveal Melanoma |
| ACSL4 | acyl-CoA synthetase long-chain family member 4 |
| ALOX15 | arachidonate 15-lipoxygenase |
| ATP5G3 | ATP synthase, H+ transporting, mitochondrial Fo complex subunit C3 |
| CARS | cysteinyl tRNA synthetase |
| CS | citrate synthase |
| DPP4 | dipeptidyl-dippeptidase-4 |
| GLS2 | glutaminase 2 |
| PCAT3 | lysophosphatidylcholine acyltransferase 3 |
| NCOA4 | nuclear receptor coactivator 4 |
| RPL8 | ribosomal protein L8 |
| TFRC | transferrin receptor |
| SAT1 | spermidine/spermine N1-acetyltransferase 1 |
| SLC1A5 | solute carrier family 1 Member 5 |
| FDFT1 | farnesyl-diphosphate farnesyltransferase 1 |
| CISD1 | CDGSH iron sulfur domain 1 |
| FANCD2 | Fanconi anemia complementation group D2 |
| GPX4 | glutathione peroxidase 4 |
| HSPB1 | heat shock protein beta 1 |
| MT1G | metallothionein-1G |
| NFE2L2 | nuclear factor, erythroid 2 like 2 |
| SLC7A11 | solute carrier family 7 member 11 |
| EMC2 | ER membrane protein complex subunit 2 |
| HSPA5 | heat shock protein family A member 5 |
| CDKN1A | cyclin-dependent kinase inhibitor 1 |
| CD274/PD-L1 | cluster of differentiation 274/Programmed cell death 1 ligand 1 |
| CTLA4 | cytotoxic T-lymphocyte antigen 4 |
| LAG3 | lymphocyte-activation gene 3 |
| PDCD1LG2 | Recombinant Programmed Cell Death Protein 1 Ligand 2 |
| SIGLEC15 | sialic acid binding Ig-like lectin 15 |
| IDO1 | indoleamine 2,3-dioxygenase 1 |
| PDCD1 | programmed cell death 1 |
| TIM-3 | T cell immunoglobulin domain and mucin domain-3 |

| Supplementary Table 2. The clinical characters of sarcoma patients in TCGA cohort. | |
| --- | --- |
| Clinical characters | Number |
| Alive | 161 |
| Dead | 99 |
| Mean (SD) | 60.6 (14.7) |
| FEMALE | 141 |
| MALE | 119 |
| ASIAN | 6 |
| BLACK | 18 |
| WHITE | 227 |
| Metastasis | 68 |
| Primary | 8 |
| Recurrence | 49 |
| Non-radiation | 140 |
| Radiation | 74 |
| Neoadjuvant | 1 |
| No neoadjuvant | 259 |
| Ancillary:Chemotherapy | 1 |
| Ancillary:Chemotherapy:Hormone Therapy | 1 |
| Chemotherapy | 59 |
| Chemotherapy: | 1 |
| Chemotherapy:Hormone Therapy | 2 |
| Chemotherapy:Immunotherapy | 3 |
| Chemotherapy:Immunotherapy:Targeted Molecular therapy | 1 |
| Chemotherapy:Targeted Molecular therapy | 3 |
| Hormone Therapy | 1 |

| Supplementary Table 3. Gene variation of 32 FRGs in SARC | | | | |
| --- | --- | --- | --- | --- |
| Clinical Attribute | Attribute Type | Statistical Test | p-Value | q-Value |
| **Mutation Count** | Sample | Wilcoxon Test | 9.081E-05 | 0.003258 |
| **TMB (nonsynonymous)** | Sample | Wilcoxon Test | 0.0001629 | 0.003258 |
| **Fraction Genome Altered** | Sample | Wilcoxon Test | 0.0004256 | 0.005674 |
| **Aneuploidy Score** | Sample | Wilcoxon Test | 0.008493 | 0.0764 |
| **MSIsensor Score** | Sample | Wilcoxon Test | 0.0103 | 0.0764 |
| **Radiation Therapy** | Patient | Chi-squared Test | 0.0115 | 0.0764 |
| **Oncotree Code** | Sample | Chi-squared Test | 0.0494 | 0.247 |
| **Cancer Type Detailed** | Sample | Chi-squared Test | 0.0494 | 0.247 |
| Subtype | Patient | Chi-squared Test | 0.0772 | 0.343 |
| Race Category | Patient | Chi-squared Test | 0.115 | 0.454 |
| Tumor Type | Sample | Chi-squared Test | 0.141 | 0.454 |
| New Neoplasm Event Post Initial Therapy Indicator | Patient | Chi-squared Test | 0.173 | 0.462 |
