# Supplementary material for: Identification of Ferroptosis-Related Genes as Biomarkers for Sarcoma
Source: Front Cell Dev Biol. 2022 Mar 1;10:847513. doi: 10.3389/fcell.2022.847513 (PMC8929291; doi:10.3389/fcell.2022.847513)
Supplement: Supplementary file 2 [file DataSheet1.docx]

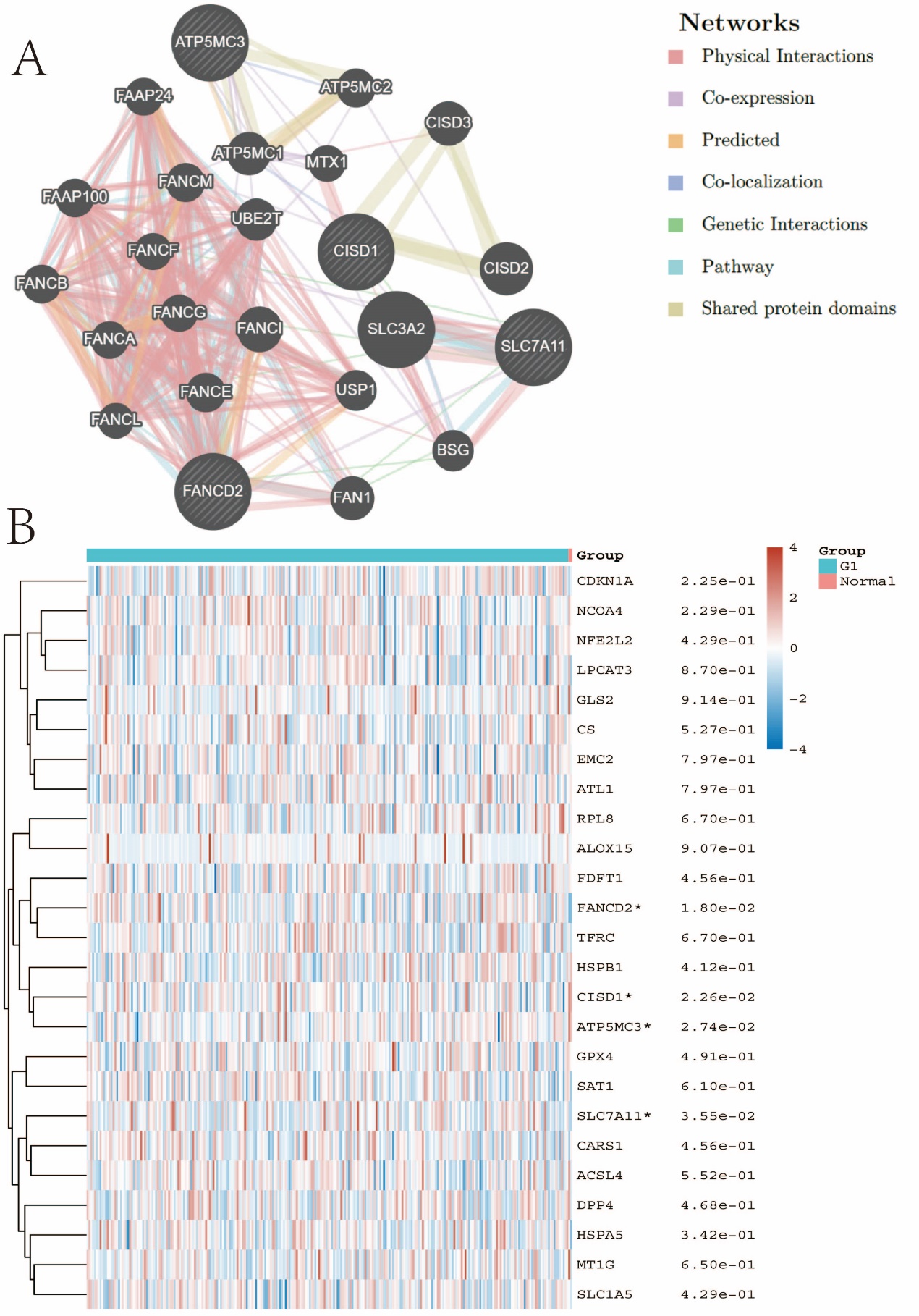


Supplement figure 1. A GeneMAMA(**A**) and protein-protein interaction(**B**) analysis and heatmap analysis between 32 FRGs gene.


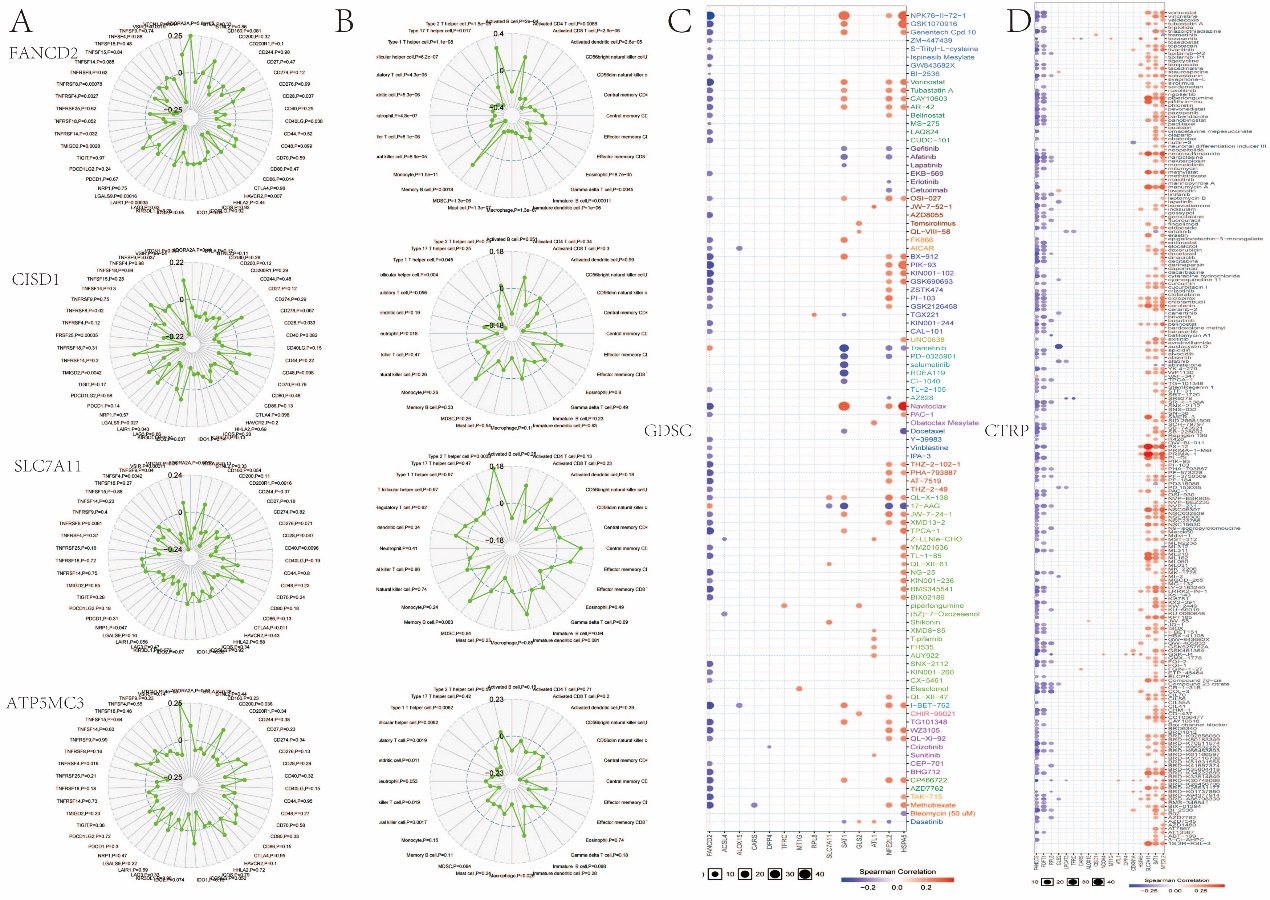


Supplement Fig. 2 immunecheck point, immune pathway, GDSC and CTRP analysis of FRG in SARC. **A** The correlation between 4 prognostic FRG and immunecheck point in SARC. **B** The correlation between 4 prognostic FRG and immune pathway in SARC. **C** The correlation between 4 prognostic FRG and GDSC in SARC. **D,** The correlation between 4 prognostic FRG and CTRP in SARC. TMB tumour mutation burden, MSI microsatellite instability, LUAD lung adenocarcinoma, FRG, Ferroptosis‑related gene, CTRP cancer therapeutics response portal. GDSC, Genomics of Drug Sensitivity in Cancer.


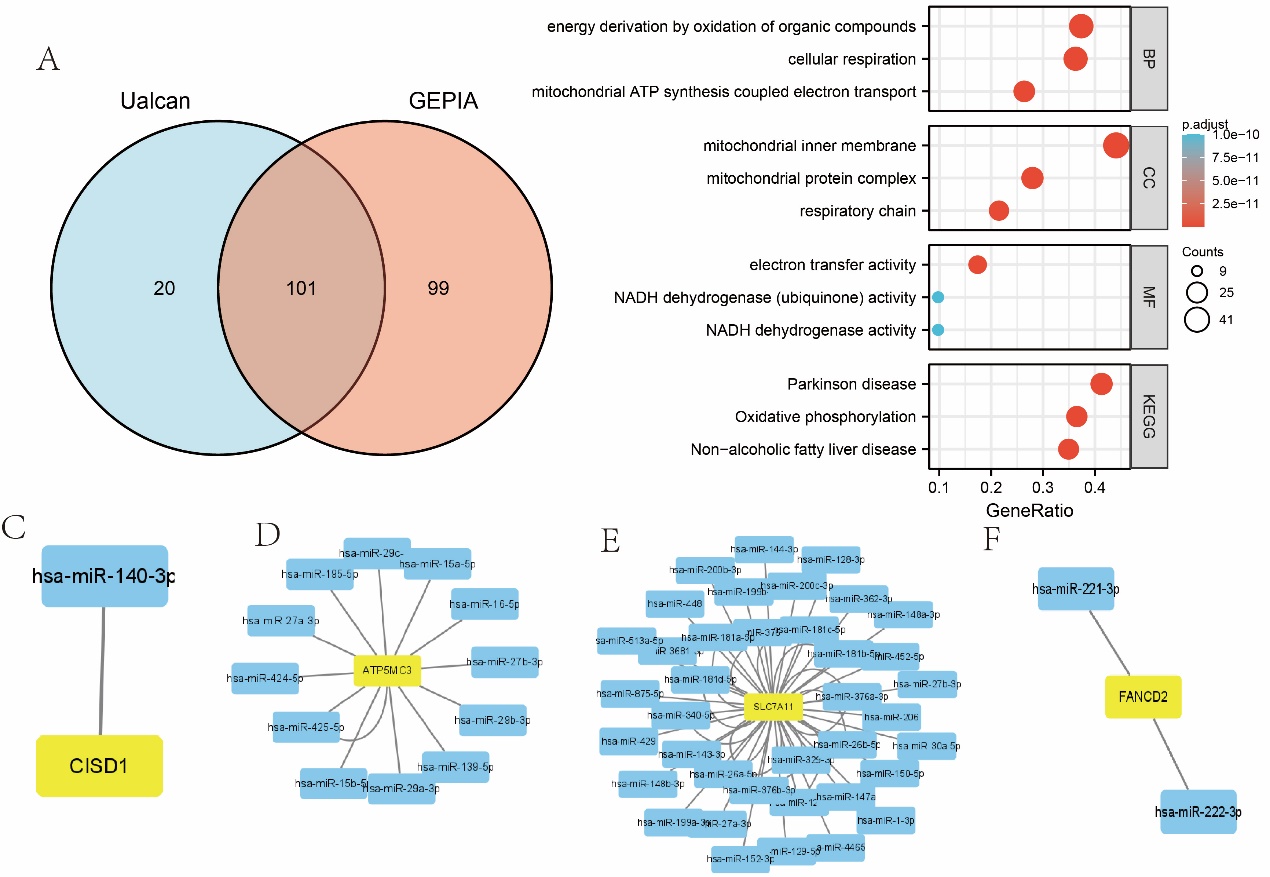


Supplement Fig. 3. Enrichment analysis of co-expressed genes of 4 prognostic FRG in SARC. (A) Identification of co-expressed genes of 4 prognostic FRG in SARC using UALCAN and GEPIA database. The top 10 enriched biological process (BP) items for the co-expressed genes of 4 prognostic FRG in SARC. The top 10 enriched cellular component (CC) items for the co-expressed genes of 4 prognostic FRG in SARC. The top 10 enriched molecular function (MF) items for the co-expressed items for the co-expressed genes of 4 prognostic FRG in SARC. The top 10 enriched KEGG items for the co-expressed items for the co-expressed genes of 4 prognostic FRG in SARC. (**B**) The miRNA-4 prognostic FRG network established by Cytoscape.


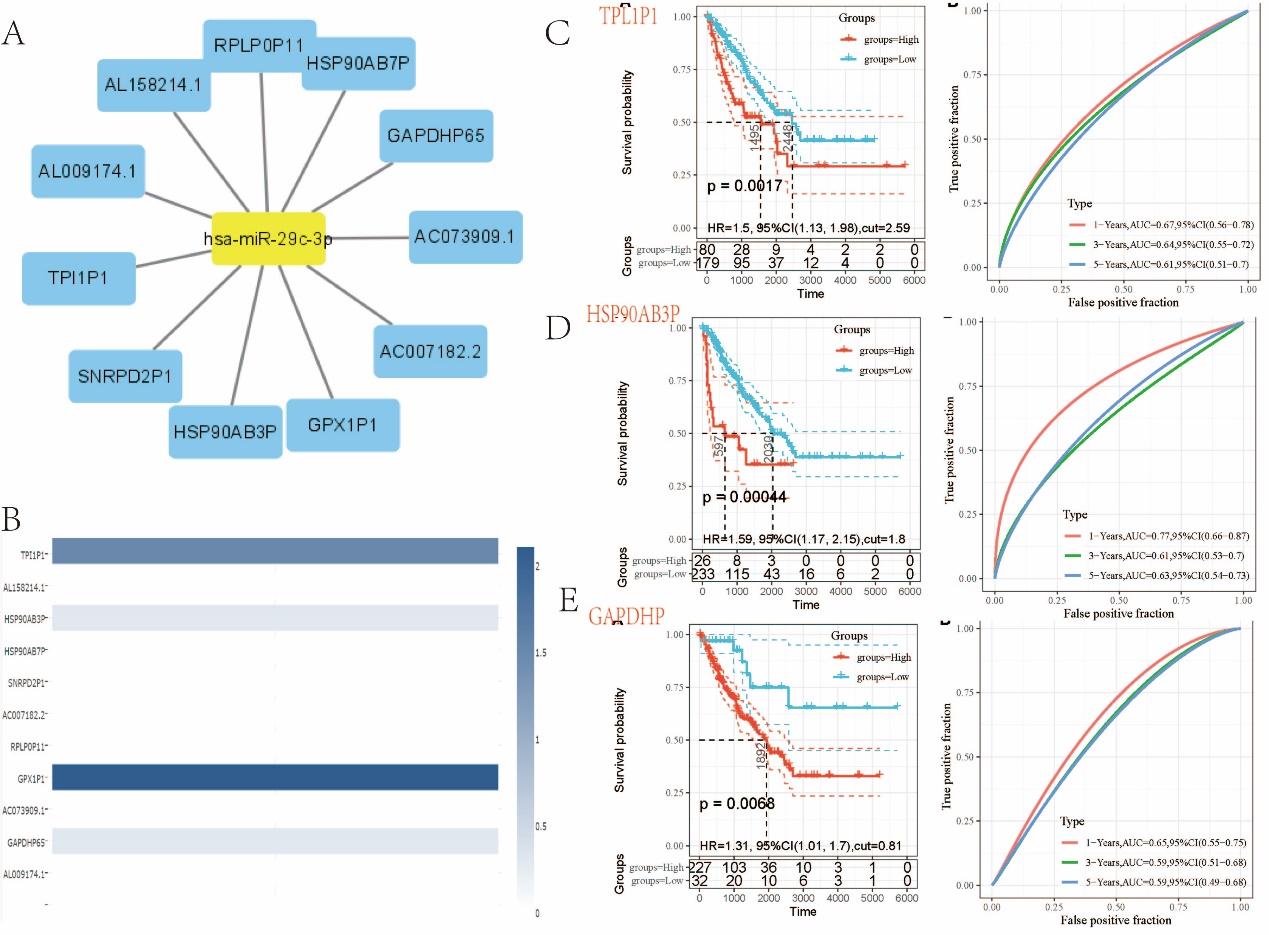


Supplement Fig. 4. Identification of upstream potential pseudogenes of hsa-miR-29C-3p in sarcoma. (A) The pseudogenes- hsa-miR-29C-3p network constructed by Cytoscape. (B) The expression levels of pseudogenes- hsa-miR-29C-3p. (C) The prognosis value of TPL1P1. (D) The prognosis value of HSP90ABP3P. (C) The prognosis value of GAPDHP.


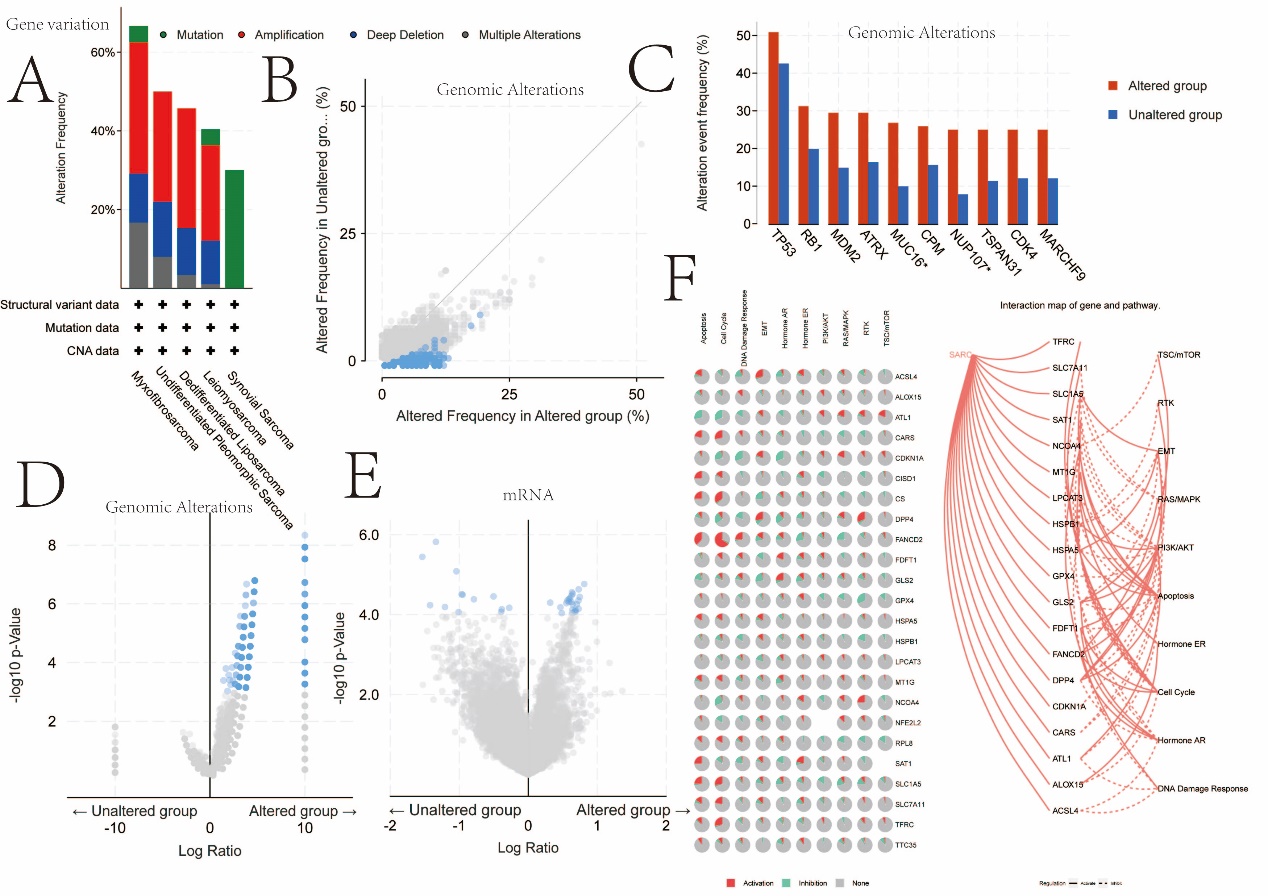


Supplement Fig. 5. Gene variation and pathway analysis in SARC. (A) Gene variation. (B) Genomic alterations. (C) 10 genes related with genomic alteration. (D,E) mRNA level of gene variation. (F) pathway analysis in SARC.


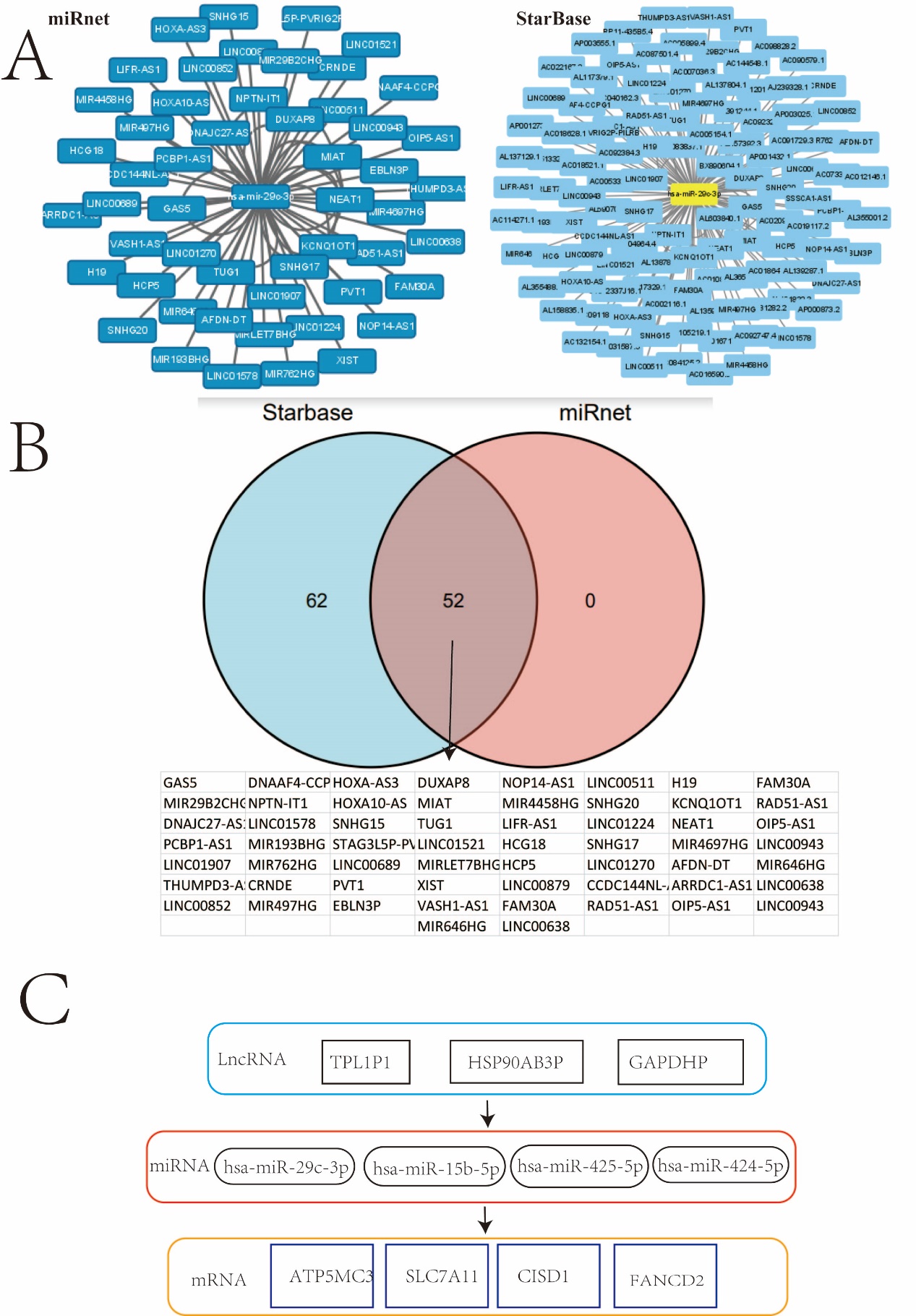


Supplement Fig. 6. Screening upstream potential lncRNAs of hsa-miR-29C-39. (A) The potential lncRNAs of hsa-miR-29C-39 predicted by starBase database and miRNet database. (B) 52 intersected lncRNAs from starBase and miRNet databases. (C) LncRNA-miRNA-mRNA network.
